# Exendin-4 Upregulates Adiponectin Level in Adipocytes via Sirt1/Foxo-1 Signaling Pathway

**DOI:** 10.1371/journal.pone.0169469

**Published:** 2017-01-25

**Authors:** Anping Wang, Ting Li, Ping An, Wenhua Yan, Hua Zheng, Baoan Wang, Yiming Mu

**Affiliations:** Department of Endocrinology, Chinese PLA General Hospital, Beijing, China; Brown University Warren Alpert Medical School, UNITED STATES

## Abstract

Glucagon-like peptide-1 (GLP-1) receptor plays an essential role in regulating glucose metabolism. GLP-1 receptor agonists have been widely used for treating diabetes and other insulin resistance-related diseases. However, mechanisms underlying the anti-diabetic effects of GLP-1 receptor agonists remain largely unknown. In this study, we investigated the effects of GLP-1 agonist exendin-4 on the expression of adiponectin, an insulin sensitizing hormone. We found that exendin-4 increased the expression and secretion of adiponectin both *in vitro* and *in vivo*. Our data showed that exendin-4 upregulated adiponectin expression at both mRNA and protein levels in adipocytes and adipose tissues. The effects of exendin-4 on adiponectin expression were dependent on the GLP-1 receptor. We further demonstrated important roles of Sirt1 and transcriptional factor Foxo-1 in mediating the function of exendin-4 in regulating adiponectin expression. Suppression of Sirt1 or Foxo-1 expression significantly impaired exendin-4-induced adiponectin expression. Consistently, exendin-4 up-regulated Sirt1 and Foxo-1 expression *in vivo*. Our work is the first study demonstrating the role of Sirt1/Foxo-1 in regulating the regulatory function of a GLP-1 receptor agonist in adiponectin expression both *in vitro* and *in vivo*. The results provide important information for the mechanism underlying the function of GLP-1R on improving insulin resistance and related diseases.

## Introduction

Exendin-4 is a natural agonist of the glucagon-like peptide 1 receptor (GLP-1R). Exendin-4 and its analogues have been used in clinic for treating diabetes [1, 2]. Exendin-4 exerts its anti-diabetic function by binding to GLP-1R and activating downstream signaling pathways [1, 2]. Due to its longer half-life comparing to glucagon-like peptide 1 (GLP), the endogenous ligand of GLP-1R, exendien-4 has better and longer-lasting effects on promoting glucose-dependent insulin secretion [1, 3, 4, 5, 6].

In addition to regulating pancreatic insulin secretion, exendin-4 also functions in other types of tissues. For example, in liver, exendin-4 treatment activates GL-1R receptor and enhances insulin action [7]. In adipose tissue, exendin-4 has been shown to improve lipid profiles and cholesterol homeostasis [8]. However, the mechanism underlying the role of exeindin-4 as an insulin sensitizer remains largely unknown.

Adiponectin is a hormone mainly secreted from the adipose tissue. This hormone plays an important role in regulating metabolism and energy homeostasis. More importantly, adiponectin functions as a potent insulin sensitizer that exerts anti-diabetic functions [9]. Adiponectin regulates glucose and fatty acid metabolism in muscle, liver, adipose tissue and other tissues through AMPK, PI-3K, and MAPK pathways [9]. In adipose tissue, adiponectin regulates adipocyte differentiation, preadipocyte proliferation, insulin sensitivity, and lipid accumulation [9]. Consistent with adiponectin’s insulin-sensitizing function, lower adiponectin level has been observed in obese and diabetic populations [10, 11], suggesting a reverse correlation between the adiponectin level and insulin sensitivity. Results from animal studies also support this notion. Adiponectin transgenic mice are protected from high fat diet-induced insulin resistance [12]. Knocking out adiponectin or its receptors results in the development of insulin resistance and glucose intolerance in mice [13].

Exendin-4 has been shown to induce adiponectin secretion in adipocytes [14]. However, the molecular mechanisms underlying this function are not clear. More importantly, it is unknown whether exendin-4 has any effect on adiponectin expression *in vivo*. In this study, we demonstrated that exendin-4 up-regulated adiponectin expression in both adipocytes and adipose tissue in mice through Sirt1/Foxo-1 signaling. Our data elucidates important mechanism underlying the effects of exendin-4 on adiponectin level both *in vitro* and *in vivo*, and shed light on new treatments of diabetes based on exendin-4/GLP-1R functions.

## Materials and Methods

### Cell culture

The culture and maintenance of 3T3-L1 adipocytes were performed as described in [15]. For exendin-4 treatment, the cells were synchronized in RPIM 1640 medium for 18 hours, and the media were replaced with serum free media containing different concentrations of exendin-4. For dose course studies, the cells were treated without or with 1.25 nM, 2.5 nM, or 5 nM of exendin-4, respectively. For time course studies, the cells were incubated with exendin-4 for 6 hours, 12 hours, 24 hours, or 48 hours, respectively.

### Cell fractionation

About 5 × 10^7^ 3T3-L1 adipocytes were collected by trypsinization and rinsed twice with PBS containing 250 mM sucrose, 0.5 mM EGTA, 5 mM HEPES (pH 7.4). The cells were then lysed by passing through a 27-gauge needle 10 times. Post-nucleus fraction was collected after centrifugation at 700 × g for 10 min, followed by 18,000 × g centrifugation for 25 min. Different layers were extracted using a 20-gauge needle and 1-ml syringe, diluted in isolation buffer, and centrifuged at 18,000 × g for 30 min. All the fractionation steps were performed at 4°C.

### Western blot analysis

Protein expression was examined by Western blot analysis. In brief, the cells were lysed in RIPA buffer and the total protein was separated by SDS-PAGE. Primary antibodies used in this study include: anti-adiponectin antibody (CST, Cat. No. 2789, dilution 1:500), anti-Foxo-1 antibody (CST, Cat. No. 2880, dilution 1:500), anti-p-Foxo-1 antibody (CST, Cat. No. 2486, dilution 1:500), anti-GLP-1R (Abcam, Cat No. ab39072, dilution 1:1000), anti-HA tag (Abcam, Cat No. ab18181, dilution 1:1000), and anti-GAPDH (CST, Cat. No. 5174, dilution 1:5000). HRP-conjugated goat-anti-rabbit secondary antibody was obtained from Tiandeyue (Cat. No.S001), and diluted by 1:20000 in the Western blot analysis. For each Western blot analysis, 3 independent experiments were carried out and representative results are shown in the figures.

### Real-time PCR (RT-PCR)

Total RNA was isolated by TRIzol®/chloroform extraction. The RNA was precipitated by isopropanol and washed with 75% ethanol. The RNA was resuspended in DEPC-treated water and the concentration was measured on a Nanodrop 2000 spectrometer. The cDNA was reverse-transcribed from the RNA template using PrimeScript™ RT reagent Kit with gDNA Eraser following the manufacturer’s instructions.

RT-PCR was performed using the cDNA on an ABI 7500 Real-time PCR system. The RT-PCR was performed by running the following program: 95°C 30 seconds followed by 45 cycles of (95°C, 5 seconds; 60°C, 40 seconds). The primers used for RT-PCR were: adiponectin Forward primer: 5’-GTATTCAGGATGCTACTGTTGC-3’, adiponectin Reverse primer 5’-CTCGAGTCAGTTGGTGTC-3’, internal control Forward primer: 5’-GCTATCCAGGCTGTGCTATC-3’, and internal control Reverse primer 5’-ACTGTGTTGGCGTACAGGTC-3’.

### Enzyme-linked immunosorbent assay (ELISA)

Culture media of 3T3-L1 adipocytes were collected for ELISA to measure the level of secreted adiponectin. ELISA assays measuring adiponectin levels in the media of 3T3-L1 cells and mouse serum were performed using adiponectin (human) ELISA Assay Kit (Biovision, Cat. No. K4901-100) and adiponectin (mouse) ELISA Assay Kit (Biovision, Cat. No. K4902-100), following manufacturer’s instructions.

### Transfection of 3T3-L1 adipocytes

3T3-L1 adipocytes (ATCC, Cat. No. CL-173) were transfected with siRNA against GLP-R (Santa Cruz, Cat. No. sc-35382), Foxo-1 (Santa Cruz, Cat. No. 45760), Sirt1 (Santa Cruz sc-40986or their scramble controls using lipo RNAiMAX reagent (GIBCO, Cat. No 13778–150). The transfected cells were collected 48 hours post-transfection for detecting the knock-down efficiency. For overexpression of GLP-1R, 3T3-T1 adipocytes were transfected with pCMV3-C-HA GLP-1R expression vector (Sino Biological Inc, Cat. No.: HG13944-CY) using Lipofectamine 2000 (Invitrogen Life Technologies) following the manufacturer’s instructions.

### Animal studies

Six-week-old C57BL/6J mice were obtained from The Jackson Laboratory and bred under standard conditions with a 12-h light/dark cycle. All procedures were approved by the medical ethics committee of the Chinese PLA General Hospital. The mice were randomly divided into 4 groups (n = 6/group) as follows: Group 1, normal chow control (10 kcal % fat, 20 kcal % protein, and 70 kcal % carbohydrate); Group 2, normal chow control plus 1 nmol/kg/day exendin-4 via intraperitoneal (IP) injection; Group 3, high fat diet (HF, 45 kcal % fat, 20 kcal % protein, and 35 kcal % carbohydrate); and Group 4, high fat diet plus 1 nmol/kg/day exendin-4 via IP injection. For Groups 2 and 3, exendin-4 was injected every other day for 10 weeks. For Groups 1 and 3, saline was injected every other day for 10 weeks. The mice had access to their specific diet and water ad libitum. At week 11, after overnight fasting, serum samples were collected and the mice were sacrificed. The adipose tissues were extracted, immediately frozen in liquid nitrogen, and stored at −80°C until RNA and protein extraction.

### Statistical analysis

Data and results were reported as means ± SEM. Statistical comparisons were performed with Student’s t-tests. Values of *p*<0.05 were considered statistically significant. “*” indicates *p*<0.05. “**” indicates *p*<0.01.

## Results

### Exendin-4 upregulated adiponectin expression

**Fig 1:**
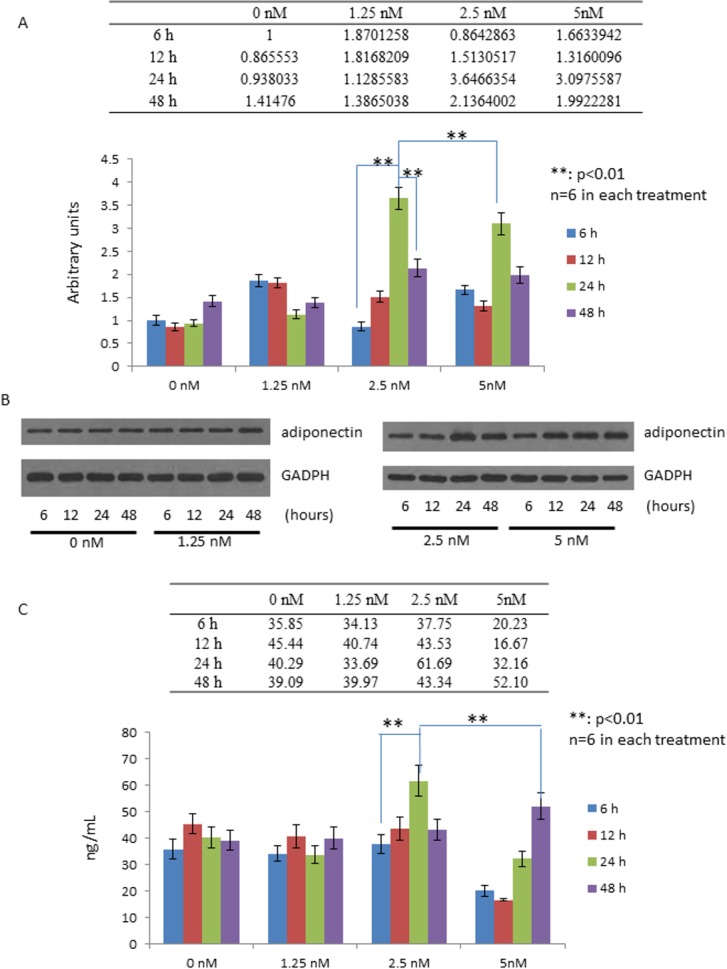
Time course and dose course studies for the effect of exendin-4 on adiponectin expression in 3T3-L1 adipocytes. (A) Effects of exendin-4 on adiponectin mRNA expression at different time points (6, 12, 24, and 48 hours) and doses (0, 1.25, 2.5, and 5 nM). (B) Effects of exendin-4 on adiponectin protein expression at different time points and doses. (C) Effects of exendin-4 on the level of secreted adiponectin at different time points and doses. Statistical analysis shows that treating the cells with 2.5 nM exendin-4 for 24 hours provided the highest adiponectin level.

Exendin-4 is a long lasting GLP-1R agonist [1, 2]. Chung et al. have shown the effect of exendin-4 on adiponectin expression [14]. However, the optimal condition under which exendin-4 upregulates adiponectin expression in 3T3-L1 adipocytes was unknown. Here, we first set out to explore the optimal timing and dosage for exendin-4 to induce adiponectin expression in adipocytes. To this end, we performed time course and dose course experiments. 3T3-L1 adipocytes were treated with exendin-4 at 0, 1.25 nM, 2.5 nM, and 5 nM for 6 hours, 12 hours, 24 hours, and 48 hours, respectively. Our results show that treating 3T3-L1 with 2.5 nM exendin-4 for 24 hours resulted in the highest mRNA expression of adiponectin (Fig 1A). Consistent with the mRNA expression, Western blot analysis demonstrate that 2.5 nM exendin-4 treatment for 24 hours also resulted in the highest protein expression of adiponectin (Fig 1B). Similarly, as shown in Fig 1C, the highest concentration of adiponectin in cell culture media was also detected in the 3T3-L1 adipocytes treated with 2.5 nM for 24 hours (Fig 1C). Interestingly, comparing to the 2.5 nM, 24 hours condition, higher exendin-4 concentration and longer incubation time caused decreased adiponectin expression and secretion, indicating the concentration and the incubation time are essential for the regulatory effect of exendin-4 on adiponectin expression. Together, our results show that treating 3T3-L1 with 2.5 nM exendin-4 for 24 hours gives the best induction of adiponectin expression and secretion.

### Suppression of GLP-1R expression impaired exendin-4-induced adiponectin expression

**Fig 2:**
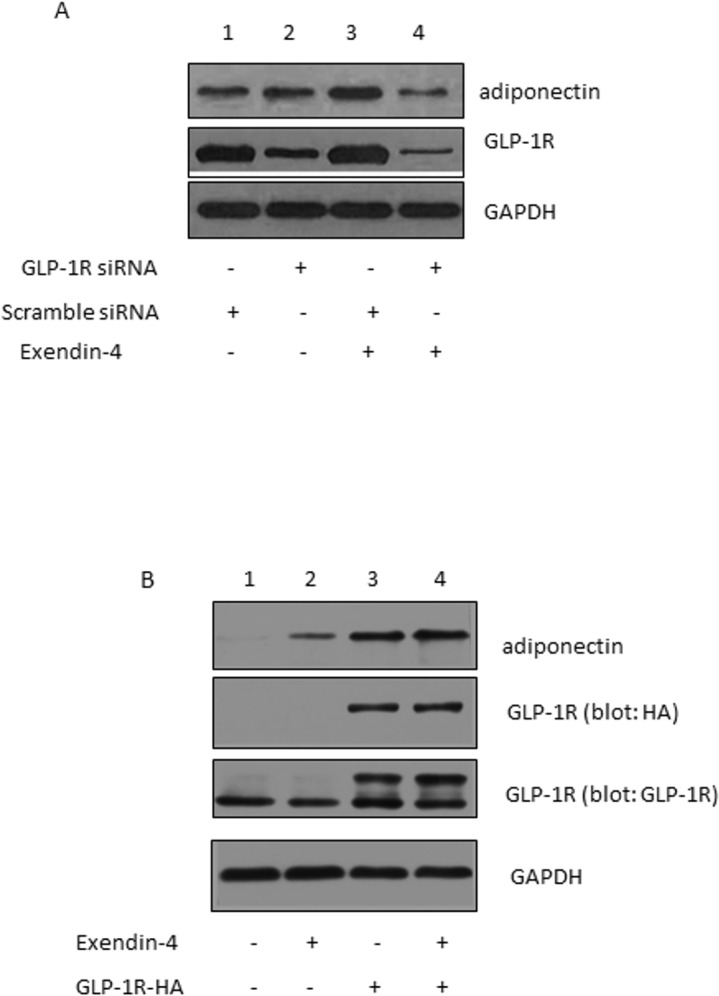
(A) GLP-1R is essential in exendin-4-upregualted adiponectin expression. GAPDH was used as an internal control showing equal protein loading. (B) Overexpression of the GLP-1R enhanced adiponectin expression.

As an agonist of GLP-1R, exendin-4 ameliorates diabetes in db/db mice through GLP-1R. [16]. In order to study the role of GLP-1R in exendin-4 induced adiponectin expression, we knocked down the expression of GLP-1R in 3T3-L1 adipocytes using siRNA targeting GLP-1R. GLP-1R expression was successfully suppressed in 3T3-L1 adipocytes that were transfected with siRNA targeting GLP-1R, while the scramble control siRNA had no effect on GLP-1R expression (Fig 2A). Exendin-4 treatment increased adiponectin expression in the scramble control cells (Fig 2A, lane 1 vs. lane 3). However, the effect of exendin-4 on adiponectin expression was significantly impaired when the GLP-1R expression was reduced (Fig 2A, lane 3 vs. lane 4). To confirm this data, we next investigated the effect of GLP-1R overexpression on adiponectin expression in adipocytes. As shown in Fig 2B, overexpression of GLP-1R enhanced adiponectin expression in adipocytes (Fig 2B, lane 1 vs. lane 3). These results demonstrate an essential role of GLP-1R in mediating exendin-4’s effect on adiponectin expression. The data indicate that upregulation of adiponectin expression is a mechanism underlying exendin-4 and GLP-1R’s role in regulating insulin sensitivity.

### Exendin-4 induced adiponectin expression through Sirt1/ Foxo-1

It has been shown that adiponectin expression is upregulated in adipocytes by the activation of Sirt1/Foxo-1 signaling [17]. Here, we tested whether adiponectin expression was induced by exendin-4 through Sirt1/Foxo-1.

**Fig 3:**
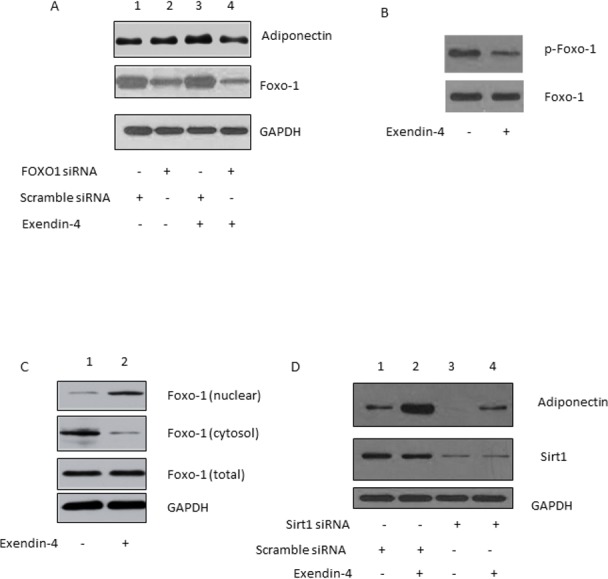
Foxo-1 and Sirt1 mediate the effect of exendin-4 on adiponectin expression. (A) Suppression of Foxo-1 expression impaired exenin-4-induced adiponectin expression. (B) Exendin-4 suppresses phosphorylation of Foxo-1. (C) Exendin-4 triggered nuclear translocation of Foxo-1. (D) Suppression of Sirt1 expression impaired exenin-4-induced adiponectin expression.

To examine the role of Foxo-1 in exendin-4 induced adiponectin expression, we knocked down Foxo-1 in 3T3-L1 adipocytes using siRNA. As shown in Fig 3A, Foxo-1 expression was successfully suppressed in 3T3-L1 adipocytes transfected with the siRNA targeting Foxo-1, while the scramble control siRNA did not have any effect on Foxo-1 expression (Fig 3, 2nd panel, lanes 1, 3 vs. lanes 2, 4). Adiponectin expression in 3T3-L1 adipocytes that were transfected with the scramble control siRNA was upregulated by exendin-4 (Fig 3A, 1st panel, lane 1 vs. lane 2). However, the effect of exendin-4 on adiponectin expression in 3T3-L1 adipocytes that were transfected with the siRNA targeting Foxo-1 was significantly impaired (Fig 3A, 1at panel, lane 3 vs. lane 4). These results indicate an essential role of Foxo-1 in regulating the effect of exendin-4 on adiponectin expression.

Foxo-1 functions as a transcription factor when it is located in the nucleus [17]. Phosphorylation causes Foxo-1 to translocate from nucleus to cytosol, thereby inactivating its transcription factor function [17]. Consistent with this mechanism, exendin-4 treatment suppressed the phosphorylation of Foxo-1 in 3T3-L1 adipocytes (Fig 3B, 1st panel). To further investigate the mechanism underlying the role of Foxo-1, we examined the translocation of Foxo-1 in response to exendin-4 treatment. We determined the Foxo-1 levels in the nuclear fraction and the cytosol fraction of adipocytes treated with or without exendin-4. The data show that exendin-4 treatment enhanced the nuclear localization, but decreased the cytosol localization of Foxo-1 (Fig 3C), indicating exendin-4 triggered Foxo-1 nuclear translocation. Together, the data suggest a role of Foxo-1 as a transcription factor mediating the effect of exendin-4 on adiponectin expression.

Sirt1 promotes Foxo-1-C/EBP transcription complex formation and increases Foxo-1-mediated adiponectin promoter activation [17]. To investigated whether exendin-4 induced adiponectin expression through Sirt1, we transfected 3T3-L1 adipocytes with siRNA against Sirt1. As shown in Fig 3D, Sirt1 expression was successfully suppressed by the siRNA, while the scramble control RNA did not affect Sirt1 expression (Fig 3D, lanes 1, 2 vs. lanes 3, 4). Adiponectin expression was induced by exendin-4 treatment in 3T3-L1 adipocytes that were transfected with the scramble control siRNA but not in the cells transfected with the siRNA targeting against (1st panel, lane 2 vs. lane 4).

Together, our results suggest that exendin-4 induces adiponectin expression through the Sirt1/Foxo-1 signaling, and Foxo-1 functions as a transcription factor mediating the effect of exendin-4 on adiponectin expression.

### Exendin-4 ameliorated high-fat-diet-induced down-regulation of adiponectin

**Fig 4:**
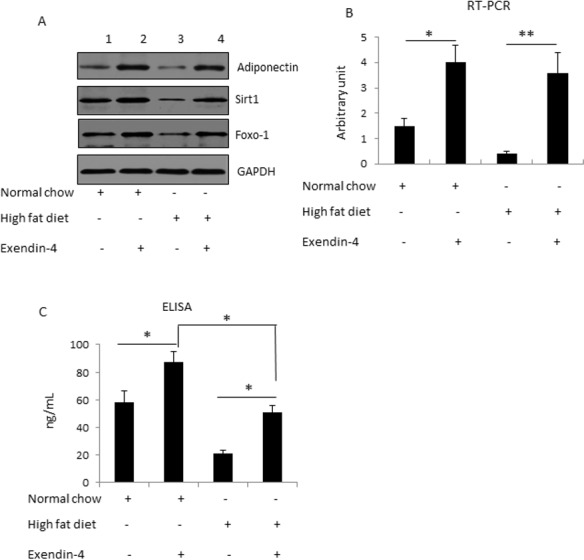
Exendin-4 promoted adiponectin expression in mice. (A) Exendin-4 promoted protein expression of adiponectin, Sirt1 and Foxo-1 in mice. (B) Exendin-4 promoted mRNA expression of adiponectin in mice. Statistical analysis shows that exendin-4 treatment significantly increased adiponectin level compared to control experiments under both normal chow and high fat diet conditions. (C) Exendin-4 promoted circulating adiponectin in mice. Statistical analysis shows that exendin-4 treatment significantly increased circulating adiponectin level compared to control experiments under both normal chow and high fat diet conditions. Also, exendin-4 treatment ameliorated but not fully reversed the circulating adiponectin level under high fat diet condition compared to the normal chow group.

Exendin-4 has been shown to function as an insulin sensitizer *in vivo* [18]. However, the underlying molecular mechanisms remain largely unknown. Downregulation of adiponectin expression in adipose tissues has been suggested as a mechanism underlying obesity-induced insulin resistance and diabetes. Thus, we investigated whether exendin-4 exerted its insulin sensitizing effect by up-regulating adiponectin. To this end, we used high fat diet-fed mice as a model for insulin resistance. Mice fed with high fat diet for 10 weeks were treated with or without exendin-4. Expression of adiponectin in adipose tissue was tested by Western blot analysis and RT-PCR. Our results show that high fat diet suppressed adiponectin expression at both protein level (Fig 4A) and mRNA level (Fig 4B). In addition, circulating adiponectin was also lowered in high fat diet-fed mice (Fig 4C). Exendin-4 treatment successfully ameliorated the high fat diet on adiponectin expression (Fig 4A and 4B) and circulating adiponectin (Fig 4C). As shown in these experiments, exendin-4 upregulated adiponectin level in mice fed with normal chow. In fact, exendin-4 significantly upregulated adiponectin expression in mice regardless the high fat diet treatment. Interestingly, although exendin-4 up-regulated adiponectin expression in adipocytes (Fig 4B), the treatment did not recover the circulating adiponectin concentration in mice fed with high fat diet to a level comparable to mice fed with normal chow (Fig 4C). This result suggests that factors other than adipose tissue expression may also regulate circulating adiponectin level. Together, these data suggest that exendin-4 plays a protective role against high fat diet-induced insulin resistance.

We next examined whether the effect of exendin-4 on adiponectin level *in vivo* was mediated by the Sirt1/Foxo-1 signaling. To this end, we tested the expression Sirt1 and Foxo-1 in adipose tissues of the mice. We found that the expression of Sirt1 and Foxo-1 were downregulated in high fat diet-fed mice (Fig 4A). Exendin-4 treatment upregulated Sirt1 and Foxo-1 levels in the high fat diet-fed mice. This result is consistent with earlier studies that show the regulatory effects of exendin-4 on Sirt1 expression and function [8].

Together, these data indicate that exendin-4 protects high fat diet-reduced adiponectin expression through the Sirt1/Foxo-1 signaling.

## Discussion

The GLP-1R agonist exendin-4 is potent in ameliorating hyperglycemia and at the same time has lower risk of causing hypoglycemia [1]. Therefore, exendin-4 has been considered as a promising treatment for diabetes and insulin resistance-related diseases [1, 4]. Exendin-4 has been shown to play important roles in promoting insulin secretion, preventing β cell apoptosis, and suppressing glucagon secretion [3–7]. However, the molecular mechanisms of exendin-4 in mediating glucose and fat metabolism remain largely unknown. Our data in the present study elucidate that exendin-4 upregulates adiponectin expression both *in vitro* and *in vivo* through the Sirt1/Foxo-1 signaling, shedding lights on molecular mechanism underlying the anti-diabetic and insulin sensitizing effect of exendin-4.

Chung et al. has shown that exendin-4 upregulates adiponectin in adipocytes [14]. However, before our study, the effect of exendin-4 on adiponectin expression *in vivo* was unknown. Moreover, no transcriptional regulatory mechanism was suggested in the effect of exendin-4 on adiponectin expression. In this study, we demonstrate that exendin-4 promotes adiponectin expression and upregulates circulating adiponectin level in mice.

More interestingly, exendin-4 treatment upregulated adiponectin levels in high fat diet-fed mice to a level significantly higher than mice fed with normal diet (Fig 4). High fat diet treatment reduces adiponectin level in mice, which has been suggested as a mechanism underlying diet-induced insulin resistance and diabetes [12, 13]. In addition, it has been reported that exendin-4 up-regulates the circulating adiponectin level in obese mice [19]. However, the mechanism underlying exendin-4’s effect on the circulating adiponectin level was unclear before this study. Our results show that exendin-4 up-regulated the circulating adiponectin level by directly regulating adiponectin expression in adipose tissues *in vivo*. We found that exendin-4 can upregulate adiponectin level regardless high fat diet treatment, suggesting that exendin-4 and high fat diet regulate adiponectin expression via different transcriptional regulatory mechanisms. Our data indicate that the transcriptional factor Foxo-1 is essential in mediating the effect of exendin-4 on adiponectin, suggesting Foxo-1 as a potential target for treating diet-induced insulin resistance and diabetes.
